# Moving beyond the mean: an analysis of faecal corticosterone metabolites shows substantial variability both within and across white-tailed deer populations

**DOI:** 10.1093/conphys/coae062

**Published:** 2024-09-07

**Authors:** Nicholas M Sutton, Cory Suski, Keegan Payne, James P O’Dwyer

**Affiliations:** Department of Biology, Grinnell College, 1116 8th Avenue, Grinnell, IA, 50112, USA; Program in Ecology, Evolution, and Conservation Biology, School of Integrative Biology, University of Illinois at Urbana-Champaign, 505 S. Goodwin Avenue, Urbana, IL, 61801, USA; Department of Natural Resources and Environmental Sciences, University of Illinois at Urbana- Champaign, 1102 S. Goodwin Avenue, Urbana, IL, 61801, USA; Department of Natural Resources and Environmental Sciences, University of Illinois at Urbana- Champaign, 1102 S. Goodwin Avenue, Urbana, IL, 61801, USA; Department of Plant Biology, University of Illinois at Urbana-Champaign, 505 S. Goodwin Avenue, Urbana, IL, 61801, USA; Carl R. Woese Institute for Genomic Biology, University of Illinois at Urbana-Champaign, 1206 West Gregory Drive, Urbana, IL,61801, USA

**Keywords:** Faecal corticosterone, glucocorticoid modelling, glucocorticoids, hormone distributions, Abbreviations: fCM, faecal corticosterone metabolite level, GC, glucocorticoid, HPA, hypothalamic–pituitary–adrenal

## Abstract

Glucocorticoid (GC) levels have significant impacts on the health and behaviour of wildlife populations and are involved in many essential body functions including circadian rhythm, stress physiology and metabolism. However, studies of GCs in wildlife often focus on estimating mean hormone levels in populations, or a subset of a population, rather than on assessing the entire distribution of hormone levels within populations. Additionally, explorations of population GC data are limited due to the tradeoff between the number of individuals included in studies and the amount of data per individual that can be collected. In this study, we explore patterns of GC level distributions in three white-tailed deer (*Odocoileus virginianus*) populations using a non-invasive, opportunistic sampling approach. GC levels were assessed by measuring faecal corticosterone metabolite levels (‘fCMs’) from deer faecal samples throughout the year. We found both population and seasonal differences in fCMs but observed similarly shaped fCM distributions in all populations. Specifically, all population fCM cumulative distributions were found to be very heavy-tailed. We developed two toy models of acute corticosterone elevation in an effort to recreate the observed heavy-tailed distributions. We found that, in all three populations, cumulative fCM distributions were better described by an assumption of large, periodic spikes in corticosterone levels every few days, as opposed to an assumption of random spikes in corticosterone levels. The analyses presented in this study demonstrate the potential for exploring population-level patterns of GC levels from random, opportunistically sampled data. When taken together with individual-focused studies of GC levels, such analyses can improve our understanding of how individual hormone production scales up to population-level patterns.

## Teaser

Hormones in wild populations can be costly to measure and difficult to describe. We used non-invasive faecal sampling to characterize distributions of glucocorticoids in three deer populations. We found significant site and seasonal differences in glucocorticoids, but similar underlying distributions, which modelling suggests are consistent with periodic spikes in glucocorticoids.

## Introduction

Glucocorticoids (GCs) are steroid hormones that are involved in a number of animal physiological processes. Classically associated with the stress response (Sapolsky *et al*., 2000; Nicolaides *et al*., 2014), GCs are also involved in a number of important physiological processes, such as modulating circadian rhythms, metabolism and generally maintaining homeostasis (Vegiopoulos and Herzig, 2007; Dickmeis, 2009; MacDougall-Shackleton *et al*., 2019). GC levels can be influenced by a number of different factors, ranging from stressors (uncontrollable events which disrupt homeostasis) like those caused by human disturbance (Creel *et al*., 2002; Taylor and Knight, 2003; Arlettaz *et al*., 2015; Tennessen *et al*., 2016; Iglesias-Merchan *et al*., 2018), predation or non-lethal predator effects (McCauley *et al*., 2011; Say-Sallaz *et al*., 2019; Allen *et al*., 2022) or weather condition (Romero *et al*., 2000; Cinque *et al*., 2021) to phenological effects like reproductive status (Romero, 2002; Geraghty and Kaufer, 2015). Such factors can induce changes in GC levels, primarily via stimulation of the sympathetic nervous system, which is responsible for the fight-or-flight response (Jansen *et al*., 1995), and via the hypothalamic–pituitary–adrenal axis (HPA axis), which is ultimately responsible for the secretion of GCs (Smith and Vale, 2006). As such, GCs are often used to monitor changes in the health and condition of animal populations. Typically, this sort of monitoring has focused on assessing changes in mean or median GC levels between sample groups or else high frequency sampling of a few individuals to detect acute changes in GC levels over a short timescale (Barja *et al*., 2007; Fredebaugh-Siller *et al*., 2013; Bryan *et al*., 2015). Such methods are invaluable, but we can gain additional insights into the processes underlying changes in GC levels through an analysis of GC level distributions in populations.

To study GCs in wildlife, biological material for hormone analysis is repeatedly collected from a number of, usually identifiable, individuals either at a high frequency over a short time scale or else at a lower frequency over longer time scales, depending on the focus of the research and whether acute or gradual changes in GCs are being measured. Individual GC responses are then expected to scale up to affect population responses (Baker *et al*., 2013), though the exact form of this scaling remains unknown. Studies of GCs in wildlife populations tend to assume that changes in GC levels are ubiquitous, or at least occur in enough individuals in the population to affect the mean GC level. However, if some extreme GC levels are only observed rarely, either due to individual variation in GC response or an infrequent stressor or phenological effect, then the mean GC level may be a poor measure of central tendency for the underlying population GC distribution. Furthermore, even populations with similar mean GC levels could still have different underlying GC distributions, and these differences could be informative. It is therefore important to consider the shape of the GC distribution in populations when comparing GC levels, and by studying the shapes of GC distributions, we also might infer the functional form connecting individual changes in GCs to population level patterns.

An exploration of GC distributions requires sampling hormones at the population level. Blood and/or saliva sampling are common invasive techniques, where direct handling of wildlife is required and may alter acute GC levels. Faecal and/or urine sampling are common noninvasive techniques, where no direct contact with wildlife is required. Both invasive and noninvasive sampling approaches provide sufficient biological material for assessing hormone levels, though each comes with its own pros and cons and will differ in temporal resolution (Sheriff *et al*., 2011). To adequately characterize the distribution of GC levels in a population, samples from a large number of individuals are needed, to the extent that it may not be feasible to track or uniquely identify every individual. Faecal hormone sampling and analysis can serve as a non-invasive and relatively low cost method for measuring GC levels in wildlife (Millspaugh and Washburn, 2004; Romano *et al*., 2010; Ralph and Tilbrook, 2016), and can enable longer-term and more widespread hormone sampling from populations if used opportunistically. While such an approach results in a loss of individual-level information, it allows for population level inferences.

In this study, we explore the distribution of GC levels in wildlife populations, as measured via faecal corticosterone metabolite levels (fCMs). Corticosterone metabolites in faeces are thought to represent a long-term measure of GC production in individuals, but they have also been shown to reliably track acute changes in circulating GC levels as well, though with some time lag. Faecal GC measures have been shown to reliably track changes in snowshoe hare (*Lepus americanus*) plasma hormone levels (Sheriff *et al*., 2010) as well as GC levels in white tailed deer (*Odocoileus virginianus*) saliva (Millspaugh *et al*., 2002). Specifically, we collected faecal samples from three white-tailed deer populations. At no point were specific deer captured, tagged, or otherwise identified. All faecal samples were collected opportunistically and cannot be matched to any particular individual. As a result, the past experiences, physical condition, and behaviour of the source of any given faecal sample remains unknown. Any such factors must therefore be inferred from the observed distributions of fCMs in these populations. To make these inferences, we explore models of individual GC level changes and their associated fCM distributions at the population level. While there are obvious drawbacks to such an approach (a lack of history on sample sources makes drawing conclusions difficult), there are several benefits as well. Anonymous, opportunistic faecal sampling of wildlife populations is low in cost and effort, allowing for greater sample size and longer sampling duration than might be possible if samples were only collected from a small, identifiable subset of the population.

Following the design described above, we collected random faecal samples from three white-tailed deer populations following an opportunistic sampling approach. We analysed faecal samples to obtain measures of corticosterone metabolites as an indicator of GC levels in the populations. We found that mean fCM levels differed between deer populations and varied seasonally. Additionally, fCM level distributions in all three populations were heavy-tailed, with the bulk of observed fCM levels being relatively small, while rare observations of fCM levels several orders of magnitude higher led to long tails. We considered two models of acute GC elevation to describe the observed heavy-tailed fCM distributions: a random GC elevation model and a periodic GC elevation model. We found that a model of periodic, acute increases in corticosterone best fit the observed fCM distributions. Finally, we discuss the significance of these findings and the potential of these sampling and modelling methods for inferring biologically relevant information surrounding changes in GC levels in wildlife populations.

## Materials and Methods

### Study sites

We collected deer faecal samples from February through November of 2019. Samples were collected from three different state parks in east-central Illinois: Kickapoo State Recreation Area (KP), Moraine View State Recreation Area (MV) and Walnut Point State Park (WP). These sites were chosen due to their differences in human activity level, with Kickapoo representing a high activity site, Moraine View an intermediate activity site, and Walnut Point a low activity site. While activity levels differ, all sites allow for similar activities (e.g. hiking, boating, camping, hunting, fishing, and cross-country skiing). Sites are far enough apart (*>* 55 km) that the deer in each site can be treated as separate populations given typical white-tailed deer dispersal distances and home range sizes in east-central Illinois (Nixon *et al*., 1991). Below we describe each site in detail. Historical park visitation data were provided by the Illinois Department of Natural Resources (Division of Parks and Recreation, Springfield IL, USA). More recent data on park visitation was not available as such data is no longer collected by the Illinois Department of Natural Resources.

Kickapoo State Recreation Area (KP; Vermilion County, IL, USA, 40.1167^°^ N, 87.7544^°^ W) is a 1150-ha park consisting of 22 deep-water ponds, a bottomland sycamore (*Platanus occidentalis*) and silver maple (*Acer saccharinum*) forest, and several areas of upland black oak (*Quercus velutina*), white oak (*Quercus alba*), sugar maple (*Acer saccharum*) and hickory (*Carya spp.*) forest. Historically, KP has the highest number of human visitors per year of the sites studied. Park attendance in 2011 was 1 541 441 visitors, and attendance was 1 124 910 visitors from September 2013 through August 2014.

Moraine View State Recreation Area (MV; McLean Co., IL, USA, 40.4109^°^ N, 88.7313^°^ W) is a 682-ha park with a 63-ha lake and several moraines covered with white and black oak, black walnut (*Juglans nigra*), sugar maple, hickory, ash (*Fraxinus spp.*) and elm (*Ulmus spp.*) trees. Park attendance in 2011 was 263 597 visitors, and attendance was 272 550 visitors from September 2013 to August 2014.

Walnut Point State Park (WP; Coles Co., IL, USA, 39.6983^°^ N, 88.0357^°^ W) is a 271-ha park with a 23-ha multi-fingered lake and woodland dominated by ash, oak, hickory, maple, walnut, black locust (*Robinia pseudoacacia*) and sassafras (*Sassafras albidum*) trees. Park attendance in 2011 was 114 317 visitors, and attendance was 198 716 visitors from September 2013 to August 2014.

### Faecal sampling

We searched for faecal deposits along deer trails, hiking trails, campgrounds and areas of parks where deer activity was observed. Upon finding deer faecal deposits, we first assessed faeces for significant environmental degradation (typically either desiccation or deliquescence, depending on the weather). We assessed degradation visually as well as via a ‘squish-test;’ if, when squished, pellets from a faecal deposit crumbled or became runny then the samples were determined to be too far degraded. Because sampling was random and the age of samples could not be directly determined, the ‘squish-test’ is meant as a conservative approach to avoid older faecal deposits and standardize pellet age across samples. This does mean, however, that naturally runny, but fresh, stools were also rejected, and only pliant, malleable pellets that could be squished without falling apart were collected. We intended our degradation assessments to constrain collected samples to those that were most likely to be recent deposits. If a faecal deposit passed our degradation assessments, then we collected several pellets from the deposit and immediately placed them in a labelled microcentrifuge tube and froze them in liquid nitrogen in the field. We then transported samples to the laboratory and stored them at −80°C until extraction.

### fCM extraction and analysis

We followed an extraction protocol similar to other studies of mammalian faecal GC metabolites (Fredebaugh-Siller *et al*., 2013; Green *et al*., 2018). First, samples were weighed to obtain wet weights and were then baked at 70°C for 16 hours. Samples were then weighed again to obtain dry weight and were then ground into a fine powder (we note here that sample refers to the several pellets taken from the same deposit, and that these pellets were homogenized when pulverizing to obtain one sample per deposit). We then weighed out 0.05 g of powdered faecal sample and mixed the sample with 1 ml of 80% ethanol and homogenized. After homogenization, samples were vortexed and then placed horizontally on an orbital shaker for 18 hours. Samples were then vortexed again and placed in a centrifuge for 30 minutes. The supernatant was then collected and stored in a labelled microcentrifuge tube at −80°C until laboratory analysis.

Faecal corticosterone metabolite concentrations were quantified via an enzyme-linked immunosorbent assay (ELISA) using donkey anti-sheep IgG with 100% corticosterone cross reactivity (and 28% deoxycorticosterone cross reactivity). While cortisol is the dominant circulating GC in white-tailed deer (Smith and Bubenik, 1990), faecal corticosterone metabolites have been shown to reliably track changes in white-tailed deer GC levels specifically (Millspaugh *et al*., 2002; Taillon and Côté, 2008; Mccoy and Ditchkoff, 2012). Additionally, the effects of environmental condition on corticosterone metabolite levels in white-tailed deer faeces are known (Washburn and Millspaugh, 2002), which enables us to better understand the potential variability in samples of unknown age, given an assessment of environmental degradation. Extractions were analysed at either a 1:100 or 1:1000 dilution (determined via serial dilutions, and showing 97% linearity) in the ELISA kit following manufacturer protocol (ADI-900-097; Enzo Life Sciences Inc., Farmingdale, NY, USA). Optical densities were obtained for each sample via microspectrophotometry and corticosterone metabolite concentrations were calculated via extrapolation from corticosterone standard curves. For plates run at a 1:1000 dilution, the inter-assay CV was 1.97% and mean intra-assay CV was 3.10%. For both dilutions, all sample duplicates fell below a CV of 15%, well within the acceptable range specified by the manufacturer.

### Statistical analyses

We tested for differences in fCM levels across sites and by month using Kruskal–Wallis tests followed by pairwise Wilcoxin rank sum tests. We then tested whether observed fCM levels followed either normal or log-normal distributions using Shapiro–Wilk goodness of fit tests. We additionally used a bootstrapping approach to determine whether observed fCM levels at each site followed power-law distributions (Clauset *et al*., 2009). Finally, we use maximum likelihood estimation with bootstrapped exact, one-sample Kolmogorov–Smirnov tests to assess the goodness of fit of two toy models of GC elevation; a random-elevation model and a periodic-elevation model. All analyses were performed at the 0.05 significance level unless otherwise stated. All analyses were performed in R version 4.3.2 (R Core Team, 2023) and using the optimx package (Nash and Varadhan, 2011; Nash, 2014) and poweRlaw package (Gillespie, 2015).

## Results

A total of 198 faecal samples were collected from February through November 2019. Sixty-seven samples had concentrations far beyond the upper limit of quantification (i.e. had fCM levels higher than what the ELISA kit could reliably quantify). Of these sixty-seven samples, 59 were re-analysed at a 1:1000 dilution, while eight were lost due to logistical reasons (a lab reorganization occurred between analyses). Seven of the re-analysed samples had concentrations beyond the upper limit of quantification, even at a 1:1000 dilution, and have been excluded from analyses. We suspect these samples could have either been contaminated or degraded such that reliably estimating their fCM levels is not feasible.

Of the remaining 183 samples analysed, 78 were collected from KP (15 in February, 18 in March, 11 in April, 2 in June, 3 in July and 29 in November), 27 from MV (8 in March, 4 in April, in June, 10 in July and 2 in August) and 78 from WP (1 in February, 10 in March, 1 in April, 25 in June, 12 in July, 2 in August and 27 in November). Not all months are represented in each site as not every sampling period resulted in the acquisition of sufficiently fresh faecal samples for inclusion in this study. Additionally, when examining the distributions of fCM levels, sample size is an important consideration. If the populations are not sufficiently sampled, then rare fCM levels may be missed when characterizing distributions. However, as we show in the results below, we find heavy-tailed fCM distributions in all sites, suggesting sufficient sample sizes to detect rare fCM levels. If anything, the true populations may have even longer tailed fCM distributions with even higher fCM levels occurring very rarely. Whether the distributions we observe are typical in wildlife populations is difficult to say as, aside from this study, characterizations of cumulative distributions in wildlife hormone studies are currently rare. Certainly the range of fCM values we observe is unusually large when compared to other studies of deer fCMs (Taillon and Côté, 2008; Jachowski *et al*., 2018) where fCM levels are typically in the tens to hundreds of nanograms per gram, as opposed to the hundreds to tens of thousands of nanograms per gram we observe. Differences in methodology (radioimmunoassay versus enzyme-linked immunosorbent assay) as well as temporal extent (one season versus entire year), however, make direct comparison with other studies difficult.

### Site differences

**Figure 1:**
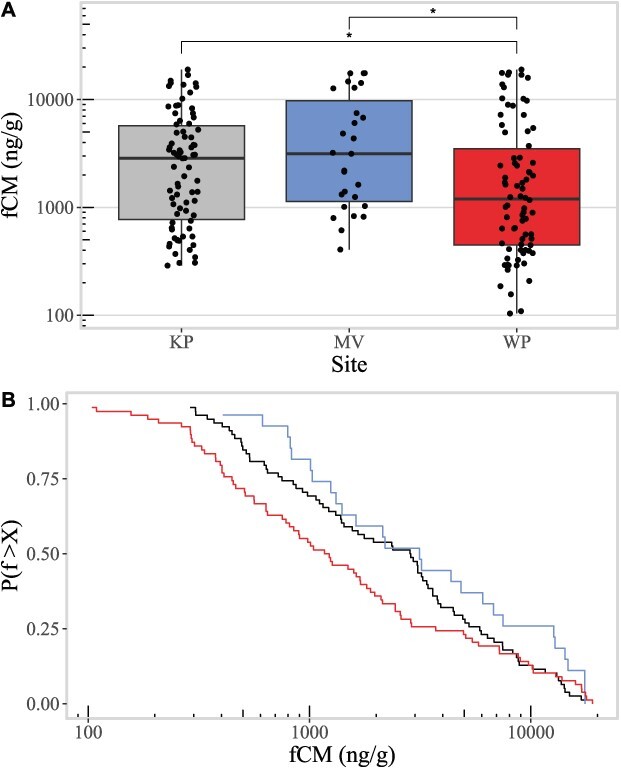
(**A**) Boxplots of faecal corticosterone metabolites (fCMs) in deer populations from Kickapoo (KP: grey, leftmost box), Moraine View (MV: blue, center box) and Walnut Point (WP: red, rightmost box). Note log scale on the y-axis. Statistically significant differences are indicated by stars above the boxes. (**B**) Solid lines—CCDFs of fCMs in deer populations from KP (black, middlemost line), MV (blue, rightmost line) and WP (red, leftmost line). Note log scale on the x-axis. The y-axis is the probability of any observed sample measurement f being greater than X.

Site had a significant effect on fCM levels (Kruskal–Wallis *P* = 0.009), and fCM levels were higher in KP and MV than in WP (Fig. 1A; KP mean fCM = 4166.53 ng/g and median fCM = 2855.84 ng/g, MV mean fCM = 5869.32 and median fCM = 3142.18, WP mean fCM = 3540.13 and median fCM = 1197.73). Differences in fCM levels between KP and MV from WP were significant, with WP fCM levels lower than both KP fCM levels (Wilcoxin rank sum *P* = 0.007) and MV fCM levels (*P* = 0.023). Levels of fCM were not significantly different between KP and MV (*P* = 0.21).

### Monthly differences

**Figure 2:**
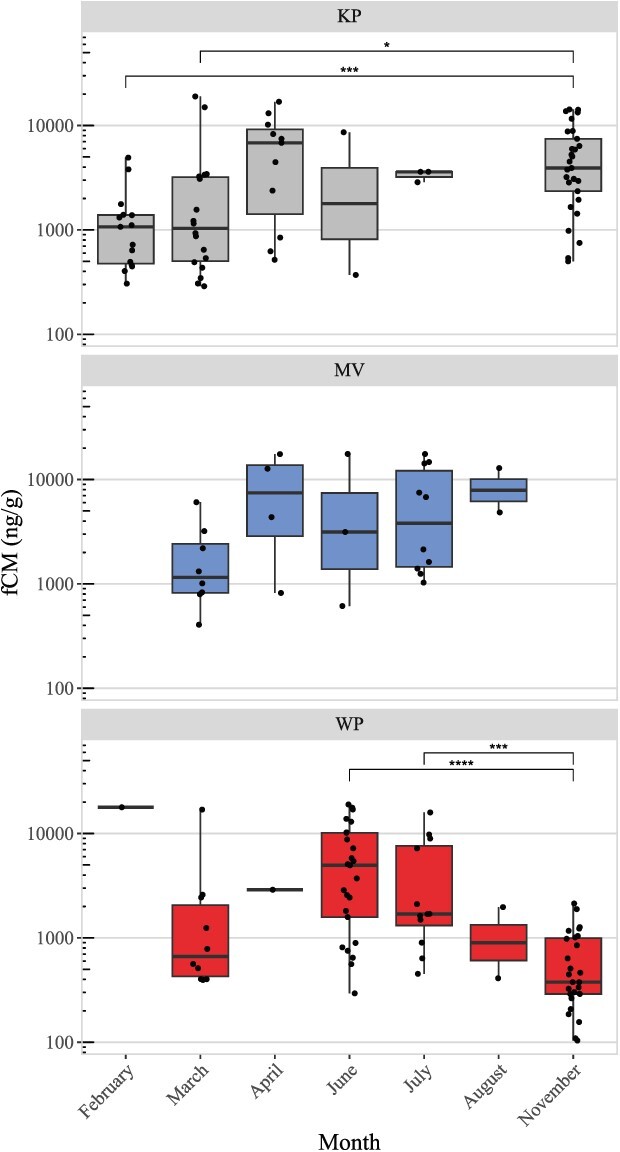
Boxplots of faecal corticosterone metabolites (fCMs) in deer populations from Kickapoo (KP: grey, top plot), Moraine View (MV: blue, middle plot) and Walnut Point (WP: red, bottom plot) per month. Note log scale on the y-axis. Statistically significant differences are indicated by stars above the boxes.

In addition to observing differences in fCM levels between sites, we also observed a significant effect of month on fCM levels in KP (Kruskal–Wallis *P* = 0.004) and WP (*P <* 0.0001), but not in MV (*P* = 0.12). In KP, fCM levels were significantly lower in November than in February (Wilcoxin rank sum *P <* 0.0001) and March (*P* = 0.004). In WP, fCM levels were significantly lower in November than in June (*P <* 0.0001) and July (*P <* 0.0001). See Fig. 2 for a full comparison of fCM levels across months within each site.

**Figure 3:**
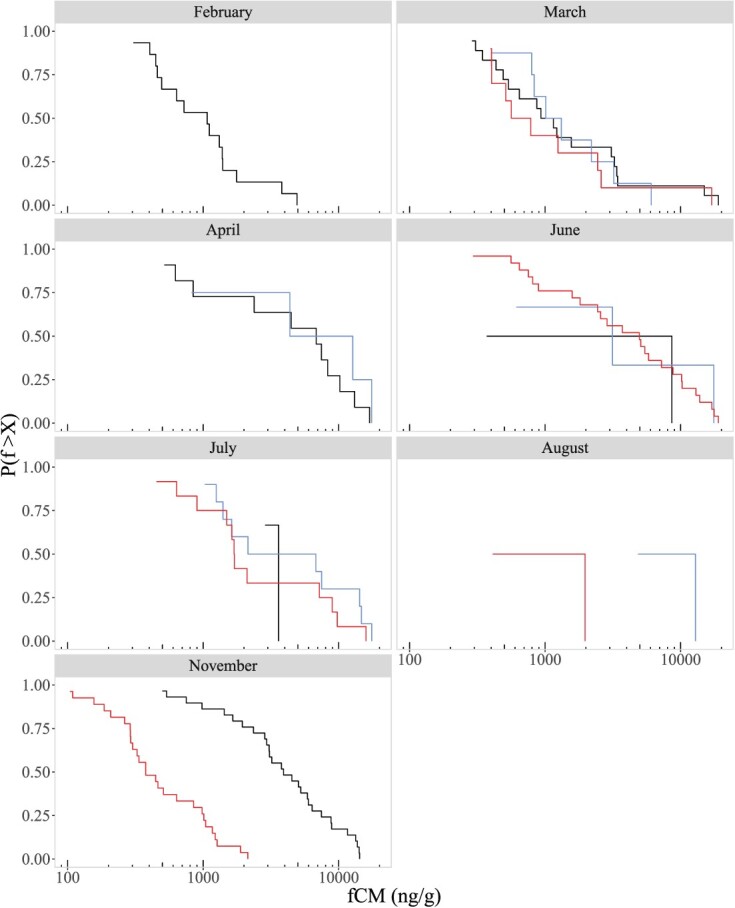
Cumulative distributions of observed faecal corticosterone metabolite (fCM) levels in deer from Kickapoo (KP: black), Moraine View (MV: blue) and Walnut Point (WP: red) by month. Note that the heavy-tailed nature of the cumulative distributions is retained when breaking data down by month, with the caveat that some months have sample sizes too low for interpreting the monthly cdf.

### fCM distributions

Levels of fCMs were broadly distributed, with very long tails similar to a power-law distribution (Fig. 1B). These long-tailed distributions were also observed when viewing distributions by month in all sites (Fig. 3). The finding of heavy-tailed fCM distributions is surprising, as one might a priori expect fCM levels to follow a normal distribution instead, given an assumption that fCM levels result from noisy, additive processes (or else follow a log-normal distribution given noisy multiplicative processes). However, when testing the goodness of fit of normal and log-normal distributions, we found that in all sites the normal distribution did not fit the observed fCM distributions (Shapiro–Wilk goodness of fit test for normal distribution; KP *P <* 0.0001, MV *P <* 0.0001, and WP *P <* 0.0001) and the log-normal distribution only offered a good fit for the MV fCM distribution, though a lower sample size in MV may mean we lack the power necessary to test this (Shapiro–Wilk goodness of fit test for log-normal distribution; KP *P* = 0.009, MV *P* = 0.057, and WP *P* = 0.01).

Instead of normally or log-normally distributed fCM levels, we found that rare instances of fCM levels orders of magnitude higher than the bulk of the data led to distributions with very long tails, similar to a power-law distribution. We therefore assessed whether a power-law distribution was a good description for the observed data via a bootstrapping approach (Clauset *et al.,* 2009; Gillespie, 2015). We found, however, that the observed fCM distributions from all sites were not well-described by a power law (KP *P* = 0.042, MV *P* = 0.016, WP *P* = 0.002; here a *P*-value greater than 0.1 means we fail to reject that the data do not follow a power law).

## Modelling the GC Response

### Are heavy-tailed fCM distributions to be expected?

The heavy-tailed distributions found in our fCM data are surprising, as one might expect fCM levels to be normally distributed around some mean population fCM level given variation in the frequency and intensity of GC elevation. One potential explanation is unknown variation in our sample quality. A drawback of our sampling method is that the exact age of faecal samples is unknown, and GC metabolites will degrade over time depending on environmental conditions. Our ‘squish-test’ was intended to minimize the impact of environmental conditions and to select for fresher samples, but we still expect some degree of degradation. A previous study examining the effect of environmental conditions and time on white-tailed deer faecal metabolites found that, in some instances, metabolite measurements would increase by upwards of 70% (Washburn and Millspaugh, 2002). While our ‘squish-test’ means we likely avoided samples more than a day old, even if we assume our measured fCM levels may vary by as much as 70%, that variation would still not account for the orders of magnitude differences we observe that lead to heavy-tailed distributions. As such, we are confident that the heavy tails we observe in white-tailed deer fCM distributions are real.

Given the heavy-tailed distributions in our fCM data, we were interested in whether such distributions are expected to naturally appear from simple models of acute GC elevation. Circulating GC levels, and subsequently GC metabolite levels in faeces, are the outcomes of complex physiological processes surrounding circadian rhythm, metabolism, stress responses and more. A complete conceptual model of GC dynamics would therefore account for any and all variation in these processes. This would include considering the distributions of GC receptor densities (for multiple receptors within the HPA axis) and their sensitivities within populations, the decay process of hormones and metabolites, attenuation or sensitization of cellular receptors, variation in diet and environment, and more. We have likely missed some important physiological processes here, but from what we have listed it should be apparent that a complete conceptual model of GC level dynamics is likely not tractable to solve. Even if it were, there remains the question of whether such complexity is necessary to describe the observed heavy-tailed fCM distributions in our data, or would a simpler model be sufficiently descriptive?

This is certainly an open question, but for now we choose to narrow our focus to two properties of GC dynamics, namely the frequency of spikes in GC levels and the rate of GC decay, that may naturally give rise to heavy-tailed fCM distributions. We consider two models of GC elevation that vary predominantly in how spikes in GC levels are distributed in time. The first is a model in which the time between successive GC spikes follows an exponential distribution, such that spikes in GCs effectively occur randomly. The second model we considered is one where GC spikes occur periodically, rather than randomly. In both models, we assume for simplicity that changes in fCM levels are directly correlated with changes in circulating GC levels, such that the fCM level observed in a faecal sample is a direct result of how close the time of defecation was to the most recent spike in circulating GC levels.

We note here that fCM levels in white-tailed deer closely match the behaviour of acute GC changes in the blood, with both a peak and exponential decay to baseline levels, but with some time lag (Millspaugh *et al*., 2002). It is possible that overlapping spikes in circulating GCs may lead to accumulation of hormone metabolites in faeces prior to deposition, or there may be some attenuation of the GC receptors such that GC levels never exceed some physiological threshold. How hormone metabolites accumulate in faeces, or if they accumulate at all during overlapping GC spikes remains unknown and is an open area of study. For simplicity, we assume no accumulation and model every GC spike as increasing circulating GC, and subsequently fCM, levels to the same value, regardless of currents levels. We know from empirical work that such an assumption is unlikely to be upheld in nature (Bell *et al*., 2007), but hold to this assumption in our modelling for the sake of reducing model complexity. Both models are described in detail below.

### Random GC elevation model

We first consider a model in which GC levels spike to a maximum, peak level before decaying to a baseline, minimum level. Here, the peak and baseline GC levels represent some physiologically constrained maximum and minimum GC levels, respectively. We then focus on the probability of measuring a given fCM level given a distribution of how long in the past the most recent GC spike occurred, combined with a relaxation time, or decay of GCs.

**Figure 4:**
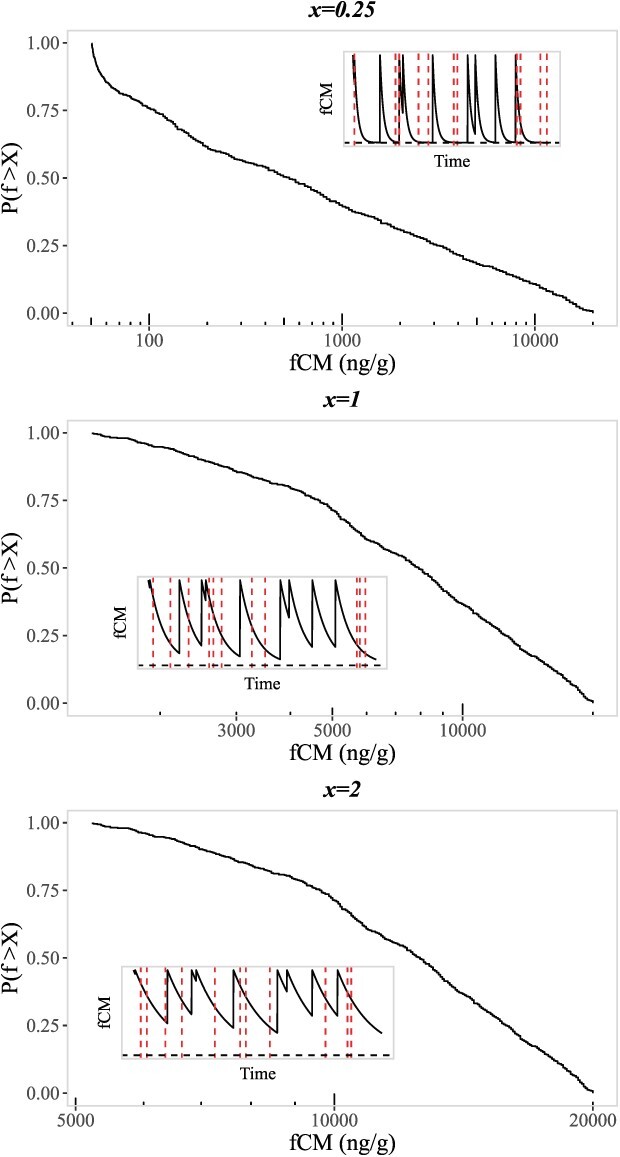
Example curves of GC dynamics (insets) for faecal corticosterone metabolite (fCM) levels given random timing of spikes. Spikes represent elevation of fCM levels, followed by exponential decay of fCM levels. Resulting cumulative distributions are shown given random sampling along the curves of GC dynamics (sampling illustrated via vertical red, dashed lines). GC dynamics and cumulative distributions are shown for three values of model parameter *x*: *x <* 1 indicates fCM spike frequency is lower than the rate of fCM decay, *x* = 1 indicates these rates are equal, and *x >* 1 indicates fCM spikes are more frequent than the rate of fCM decay.

Assuming that spikes in GC levels occur randomly and that GC levels decay exponentially, then when considering the most recent GC spike, there is a timescale (set by a rate *λ*) that characterizes the distribution of how far back one needs to go from a hormone sample measurement time to find the most recent GC spike. This involves the following assumptions:

- Waiting time is exponentially distributed with rate *λ*, essentially an assumption that GC spikes are Poisson distributed in time.
- Relaxation from peak (*f*_0_) to baseline (*f_b_*) GC level is a deterministic exponential function with rate *γ* (i.e. following a spike in corticosterone levels, the corticosterone metabolite concentration that we observe in a faecal sample is equal to *f_b_* + (*f*_0_ − *f_b_*)*e*^−*γt*^).

We then compute the generating function for the probability distribution *P*(*f*) that we observe a given fCM level *f* in a faecal sample:


(1) \begin{equation*} P(f)=x{\left(f-{f}_b\right)}^{x-1}{\left({f}_0-{f}_b\right)}^{-x} \end{equation*}

where *f* can take values between *f_b_* and *f*_0_ only, and the parameter *x* = $\frac{\lambda }{\gamma }$. Intuition around this parameter is as follows: when *λ* is small relative to *γ*, then the typical fCM measurement is relatively close to *f_b_*—though still has a fat tail all the way up to *f*_0_. When *λ* = *γ*, the distribution is uniform between *f_b_* and *f*_0_. For large *λ* relative to *γ*, the typical fCM measurement is strongly peaked towards *f*_0_. (i.e. a typical measurement will be from a GC spike in the very recent past), and corticosterone levels will not have had the chance to decline very much. See Fig. 4 for examples of GC spike dynamics consistent with this random GC elevation model, and the associated cumulative fCM distributions that result from the model GC dynamics for different values of *x*.

### Periodic GC elevation model

We next consider a model of periodic, or cyclic, GC spikes. We were interested in exploring a simple, periodic model as such a model is consistent with cyclical GC patterns like those associated with circadian rhythm. We begin with an assumption that spikes in GCs are non-overlapping, and that GC levels always return to baseline immediately prior to the next GC spike. We now assume that these spikes in GCs are regular events that occur every time interval *T*, rather than being exponentially distributed in time. This involves the following assumptions:

- The probability of collecting a faecal sample time *t* since the last GC spike is a uniform probability *p*(*t*) = $\frac{1}{T}$, where *T* is the interval between spikes. This interval *T* is related to peak and baseline GC levels and rate of decay of GCs as follows: *T* = $\frac{1}{\gamma }$log $\left(\frac{f_0}{f_b}\right)$.
- GC levels decay from peak (*f*_0_) at the start of each cycle and reach baseline (*f_b_*) at the end with rate *γ* such that the level of fCM *f* that we observe in a faecal sample deposited some time *t* from the last GC spike is given by: *f* (*t*) = *f*_0_*e*^−*γt*^.

The probability distribution *P*(*f*) that we observe a given fCM level *f* in a faecal sample is then:


(2) \begin{align*} {\displaystyle \begin{array}{l}\mathrm{P}\left(\ \mathrm{f}\ \right)=\mathrm{P}\ \left(\mathrm{t}\ \left(\ \mathrm{f}\ \right)\right)\mid \frac{dt}{df}\mid \\{}={\left(\mathrm{t}\gamma \mathrm{f}\right)}^{-1}\\{}={\left(f\log \left(\frac{f0}{fb}\right)\right)}^{-1}\end{array}} \end{align*}

**Figure 5:**
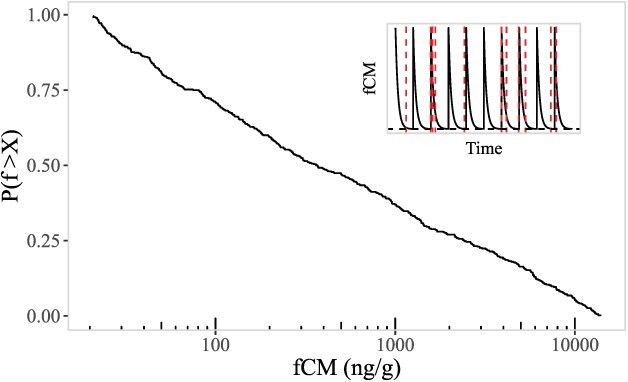
Example curve of GC dynamics for periodic spikes in fCMs (inset) and the associated cumulative distribution obtained via sampling along the curve of GC dynamics (sampling illustrated via vertical red, dashed lines).

See Fig. 5 for an example of GC spike dynamics consistent with this periodic model, and the associated cumulative fCM distribution that results from the model GC dynamics.

### Model results

In the periodic elevation model, we estimated the decay rate of corticosterone *γ* = 0.05 from experimental adrenocorticotropic hormone (ACTH) challenges in white-tailed deer (Millspaugh *et al*., 2002). In the random elevation model, *γ* = 1 so that the parameter *x* = $\frac{\lambda }{\gamma }$, which characterizes the shape of the predicted cumulative distribution, is determined entirely by the frequency of corticosterone elevation *λ*. In both models, *γ* is held constant for all populations as we assume the rate of decay of corticosterone is well-constrained within species based on some physiological and chemical constraints. Additionally, we used the minimum and maximum observed empirical fCM levels in each population as a best guess when setting baseline and peak parameter levels for testing model goodness of fit. This is based on an assumption that each population was sufficiently sampled such that we observed fCM levels very near to the minimum and maximum levels possible in white-tailed deer.

**Figure 6:**
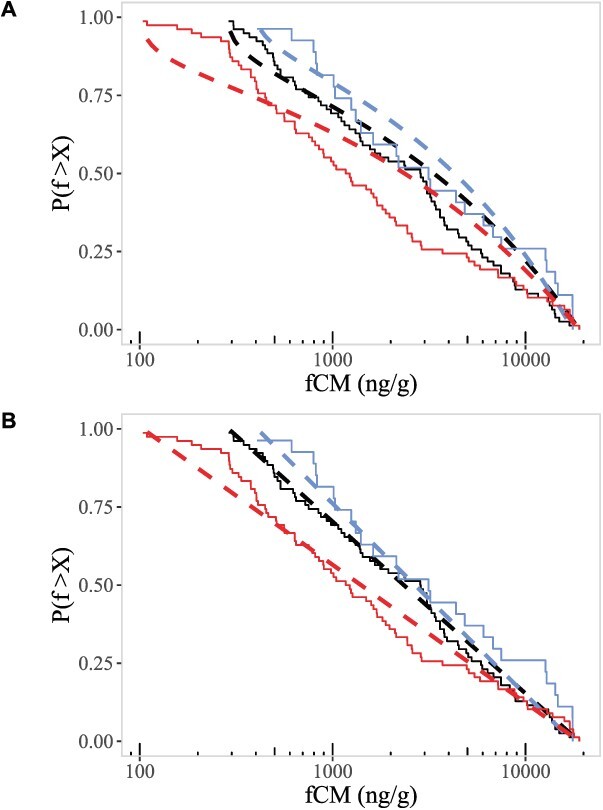
(**A**) Random GC elevation model fit (dashed lines) to observed cumulative distributions (step functions) of faecal corticosterone metabolite (fCM) levels in deer from Kickapoo (KP: black, middlemost lines), Moraine View (MV: blue, rightmost lines) and Walnut Point (WP: red, leftmost lines) (**B**) Periodic GC elevation model fit. All periodic models pass a goodness of fit test. The random model is only a good fit for MV.

We used maximum likelihood estimation to fit the random GC elevation model to observed fCM levels and then used a bootstrapped, one-sample Kolmogorov–Smirnov test to evaluate the goodness of fit of our predicted cumulative distributions, following the approach used when estimating the goodness of fit of power-law distributions (Clauset *et al*., 2009). We followed this bootstrap approach because the Kolmogorov–Smirnov test alone is not appropriate when the hypothesized distribution parameters have been estimated from the data. Here, if *P >* 0.1 we fail to reject the null hypothesis that the observed fGC data are drawn from the model predicted distributions and conclude that the model offers a good fit to the data. We found that the random GC elevation model was a good fit to the observed fCM levels from MV (*D* = 0.13, *P* = 0.66), but not KP (*D* = 0.15, *P* = 0.03) or WP (*D* = 0.21, *P* = 0.0003). All random elevation model fits are shown in Fig. 6A.

We fit the periodic GC elevation model by directly calculating the time interval *T* given the ratio of peak to baseline fCM levels, $\frac{f_0}{f_b}$. A bootstrapped, one-sample Kolmogorov–Smirnov test was again used to test the goodness of fit of the periodic model predicted cumulative distributions. Unlike the random elevation model, we found that the periodic GC elevation model was a good fit for fCM data from all sites; KP (*D* = 0.06, *P* = 0.99), MV (*D* = 0.17, *P* = 0.68) and WP (*D* = 0.12, *P* = 0.76). All periodic elevation model fits are shown in Fig. 6B.

For the random elevation model, we found all sites had similar inferred shape parameters less than one (KP *x* = 0.38; MV *x* = 0.47 and WP *x* = 0.33). This suggests that, at least in MV where the random model was a good fit, spikes in GCs tend to not overlap in time, and GC levels typically return to minimum/baseline levels prior to new GC spikes. For the periodic elevation model, we found that the calculated interval time between GC spikes was 83.72 hours in KP, 75.34 hours in MV, and 104.18 hours in WP. In all cases, and if the model were strictly true, this suggests that spikes in GC levels occur approximately every 3–4 days.

Finally, because baseline and peak GC levels may vary over time, we also tested the goodness of fit of the periodic model for each site by season. We did not test the random model by season, which is fit by maximum likelihood estimation, due to low samples sizes in many seasons, and we note that not all seasons are represented in every site. We found that the periodic model was a good fit for fCM data in all seasons and sites (KP Spring *P* = 0.89, Summer *P* = 0.24, Autumn *P* = 0.22, and Winter *P* = 0.45; MV Spring *P* = 0.86 and Summer *P* = 0.51; WP Spring *P* = 0.13, Summer *P* = 0.83 and Autumn *P* = 0.94). The estimated periodicity of spikes in GCs also differed by season (KP Spring *T* = 83.7, Summer *T* = 63.0, Autumn *T* = 67.0 and Winter *T* = 55.6 hours; MV Spring *T* = 75.3 and Summer *T* = 67.1 hours; WP Spring *T* = 75.2, Summer *T* = 83.3 and Autumn *T* = 60.5 hours).

## Discussion

Understanding wildlife health and physiological status at the population level is an important goal for the monitoring and management of wildlife populations. Traditionally, monitoring wildlife health involves repeated measures and observations sampled from a small number of known individuals and can be quite invasive. Faecal GC metabolite assessments offer one non-invasive approach to obtaining measures of condition from wildlife (Sheriff *et al*., 2011). Many studies of wildlife health utilizing faecal sampling have focused on the relationship between faecal metabolite levels and individual-specific quantities like acute stress or behavioural response to some stimulus (Creel *et al*., 2002), breeding status or physical condition (Creel *et al.*, 2009; Wey *et al*., 2015), or animal movements and habitat use (Jachowski *et al*., 2012). Less common are attempts to characterize population-level metrics of condition, and the studies that do are focused on mean GC levels rather than characterizing the full distribution of GCs within populations. This is largely due to both the many obvious benefits associated with monitoring known animals as well as the assumption that patterns of GC dynamics at the individual level will scale-up to the population level (Baker *et al*., 2013). Of interest to us is the potential to discover biologically meaningful patterns of GC levels in population data sans information surrounding individual animals (which may be unavailable in some data sets, or else are not feasible to collect).

### Site differences

To this end, we explored population trends in GC levels from faecal samples opportunistically collected from three white-tailed deer populations in east-central Illinois. We first looked for differences in GC level distributions among sites, as measured via faecal corticosterone metabolites (fCMs). We found that fCM levels were significantly higher in deer from Kickapoo State Recreation Area (KP) and Moraine View State Recreation Area (MV) than in Walnut Point State Park (WP). Additionally, all fCM level distributions in all sites were very heavy-tailed, though none were well described by a power law. Possible explanations for site differences in fCM levels are varied and include differences in food availability, disease occurrence, frequency of extreme weather events, et cetera. In all sites, differences in hunting effort by humans and coyotes could drive patterns of fCMs. Hunting is allowed in all sites, but the number of permits issued to hunters may differ between sites, leading to differences in human-induced stress. And while predation of deer in east-central Illinois has been historically low relative to other sources of mortality and limited to predation of fawns by coyotes (Nelson and Woolf, 1987; Anderson *et al*., 2015), coyotes have been shown to avoid habitat with high human activity (Gosselink *et al*., 2003) and may be more or less present in some sites.

Another possible explanation for site differences in fCM levels could be related to differences in human visitation rates at each site. KP is a park with historically high rates of human activity, with human activity levels orders of magnitude higher than in WP, with MV human activity levels lying somewhere in the middle (unpublished park attendance data provided by the Illinois Department of Natural Resources, Division of Parks and Recreation, Springfield IL, USA). In previous studies, we found that deer in KP had lower flight-initiation distances (the distance between deer and an approaching threat when deer flee) in response to an approaching human than deer in WP, with MV again lying between the other two (Sutton and Heske, 2017; Sutton and O’Dwyer, 2018). The behavioural and human activity data in these studies, however, was collected 3–6 years prior to the faecal samples collected in the present study and may no longer reflect the status of these state parks or the behaviour of their deer populations. However, if we assume that human activity and deer behaviour in 2019 was consistent with historical data, then it would appear that deer flight behaviour in response to human approaches may be inversely related to fCM levels in these populations, such that deer in KP allow humans closer but experience higher fCM levels, and vice-versa in WP. This could indicate that deer are coping when they adjust their flight response to human activity, rather than habituating, and may have chronic changes in GC levels induced by human activity. Additional research with concurrently measured flight behaviour and fCMs would be needed to verify this pattern, however.

### Monthly differences

In addition to finding differences in fCM levels between populations, we also found significant differences in fCM levels across months in KP and WP, but not in MV (though we likely lacked sufficient sample size in MV to detect significant differences). In all sites we noticed a trend towards increasing fCM levels in mid spring through early summer (April–June). In KP and MV, fCM levels appeared to remain elevated through the fall, and in KP fCM levels were at their lowest in late winter and early spring. In WP, however, fCM levels appeared to decline earlier than in KP and MV, declining significantly in the fall. These trends are consistent with corticosterone seasonal trends observed in elk (*Cervus canadensis*), where elk corticosterone declined during fall and winter, and increased through spring and summer in both males and females (Millspaugh *et al*., 2001). We considered that, given seasonal variation in fCM levels, uneven sampling of different months could account for the heavy-tailed fCM distributions we observed in all sites. When broken down by month, however, fCM distributions remained heavy-tailed (Fig. 3), suggesting that rare occurrences of very high fCM levels are likely at any time of year. This would be consistent with the Reactive Scope Model (Romero *et al*., 2009), where the bulk of observed fCM levels could represent the predictive homeostatic range of GCs in deer (i.e. normal variation in GCs due to predictable changes in environment), while the rarer, high fCM levels found in the heavy tails could represent the reactive homeostatic range of GCs in response to random stressors (i.e. elevated GCs in response to unpredictable changes in environment) or even homeostatic overload if extremely high fCM levels are associated with disease, starvation, or chronic stress.

Without additional data, we can only speculate on the mechanisms underlying the seasonal trends in our data, but are reasonably certain that the white-tailed deer breeding cycle is a contributing factor. The increase in fCM levels detected in April–July coincide with the expected parturition and lactation date ranges for white-tailed deer (May 15–July 15) (Nixon *et al*., 1991), and parturition and lactation are known to significantly increase GC levels both in deer (DelGiudice *et al*., 1992) as well as in other mammals (Kenagy *et al*., 1999; Kenagy and Place, 2000). Another potential explanation for the observed seasonal trend in fCM levels could be that fCM levels track monthly changes in human activity, as human activity level at all sites have historically been much higher in the late-spring and summer months (unpublished park attendance data provided by the Illinois Department of Natural Resources, Division of Parks and Recreation, Springfield IL, USA).

### Toy GC models

We considered two models of individual spikes in GC levels with differing timing to see if heavy- tailed fCM cumulative distributions might naturally arise from simple descriptions of GC level dynamics. Both models are based on the exponential decay of GCs immediately following a spike in hormone levels, as motivated by ACTH challenges in white-tailed deer (Millspaugh *et al*., 2002). The first model assumed spikes in GCs occur randomly in time, whereas the second model assumed spikes in GCs occurred periodically at some constant time interval, with the timing between GC spikes being characterized by the peak/maximum and baseline/minimum GC levels such that levels always returned to baseline immediately prior to the next GC spike. We found that the observed fCM distributions in all sites were best described by the model of periodic GC dynamics, with spikes in GCs expected to occur every three to four days. The longest estimated period was in WP, with GC spikes expected every 104.18 hours, a day or more longer between spikes than estimated in KP and MV. Differences in the estimated periods of GC spikes between sites might be explained by phenological differences in the deer populations, which might further explain, in part, seasonal differences in fCMs between sites. In all sites, we found that the estimated periodicity of GC spikes varied by season, and we specifically found that periods were longest in the Summer for all three sites, and shortest in Autumn or Winter. These shifting periodicities could be indicative of changing baseline GC levels throughout the year.

True GC dynamics in deer are far more complicated than our models might suggest. Habituation or attenuation of baseline GC levels and the acute response due to repeated stressors (Rich and Romero, 2005; Cyr *et al*., 2007; Cyr and Romero, 2007), potential accumulation of GC metabolites in faeces prior to deposition, and variable magnitudes of GC spikes due to idiosyncratic differences in individual physiology are just some examples of processes our modelling ignores. Adding this additional complexity to models of GC dynamics could lead to a better understanding of the mechanisms underlying the heavy-tailed fCM distributions we observe in deer. Indeed, this is an avenue of modelling individual GC dynamics that is ripe for further exploration and analysis. Our toy models, however, do demonstrate that heavy-tailed fCM distributions can naturally arise from very simple descriptions of GC dynamics, and may be driven by periodic processes. Whether heavy-tailed distributions are ubiquitous when exploring more complex models of GC dynamics, or when exploring other populations and taxa, remains to be seen, and we encourage others to examine the cumulative distributions of hormone levels where the data is available to do so.

## Conclusion

Additional information will always be useful when studying GC levels in wildlife, and the identification and monitoring of individuals is often necessary to fully understand observed changes in hormone levels. However, when such information is unavailable or the constraints of a study preclude its collection, what is one to do? We demonstrate here that a top-down analysis of wildlife GC levels, starting from population hormone measurements and moving to individual GC modelling, can be a non-invasive and non-intensive approach to studying GC dynamics in the wild. We were able to identify significant differences in fCM levels between populations, seasonal trends in fCMs, and infer information on the timing of spikes in GC levels from random, opportunistically gathered faecal samples in combination with toy models of GC dynamics. While we cannot be as conclusive as we might be with additional information, the patterns of GC levels we detected are nonetheless informative when characterizing the physiological condition of different wildlife populations.

## Data Availability

The data and code underlying this article will be shared on reasonable request to the corresponding author.
